# Case Report: Severe Hypotonia Without Hyperphenylalaninemia Caused by a Homozygous *GCH1* Variant: A Case Report and Literature Review

**DOI:** 10.3389/fgene.2022.929069

**Published:** 2022-07-13

**Authors:** Yun Chen, Kaiyu Liu, Zailan Yang, Yaozhou Wang, Hao Zhou

**Affiliations:** Department of Pediatric Neurology, Guizhou Provincial People’s Hospital, Guizhou University, Guiyang, China

**Keywords:** GCH1, dopa-responsive dystonia, hypotonia, homozygous, variant

## Abstract

Dopa-responsive dystonia (DRD) comprises a group of rare but treatable dystonias that exhibit diurnal fluctuation. The *GCH1* gene encodes GTP cyclohydrolase-1 (GTPCH-І), a protein that catalyzes the first rate-limiting step of tetrahydrobiopterin biosynthesis. Pathogenic variants in *GCH1* are the most common causes of DRD. However, the autosomal recessive form of DRD caused by biallelic *GCH1* variants is very rare. Homozygous *GCH1* variants have been associated with two clinically distinct human diseases: hyperphenylalaninemia, and DRD with or without hyperphenylalaninemia. Here, we describe one patient who presented during infancy with severe truncal hypotonia and motor developmental regression but without diurnal fluctuation and hyperphenylalaninemia. Treatment with levodopa/carbidopa resulted in the complete and persistent remission of clinical symptoms without any side effects. This was accompanied by age-appropriate neurological development during follow-up. A homozygous *GCH1* variant (c.604G>A/p.V202I) was identified in the patient. To our knowledge, this is the first Chinese case of DRD caused by a homozygous *GCH1* variant, thus expanding the spectrum of DRD phenotypes. Autosomal recessive DRD that is associated with homozygous *GCH1* variants should be considered in patients with severe truncal hypotonia, with or without diurnal fluctuation, even if there is an absence of limb dystonia and hyperphenylalaninemia.

## Introduction

Dopa-responsive dystonia (DRD), a condition first described in 1971, comprises a group of rare disorders that are clinically and genetically heterogeneous (Wijemanne and Jankovic, 2015). The typical characteristic of DRD is the childhood onset of dystonia with diurnal fluctuation; in most patients, treatment with levodopa typically results in a dramatic effect (Kim et al., 2016; Jinnah et al., 2018). Several genes have been frequently associated with DRD, including guanosine triphosphate (GTP), cyclohydrolase-1 (*GCH1*), tyrosine hydroxylase (*TH*), 6-pyruvoyl tetrahydrobiopterin synthase (*PTS*), sepiapterin reductase (*SPR*), and quinoid dihydropteridine reductase (*QDPR*) (Wijemanne and Jankovic, 2015; Weissbach et al., 2021). All of the enzymes encoded by these genes are known to be involved in the biosynthesis of monoaminergic neurotransmitters (Wijemanne and Jankovic, 2015).

The *GCH1* gene consists of six exons, is located on chromosome 14q22.1-q22.2, and encodes the GTP cyclohydrolase-1 protein (GTPCH-1). GTPCH-1 is the first rate-limiting enzyme for the biosynthesis of tetrahydrobiopterin (BH4), an essential cofactor for phenylalanine hydroxylase, tyrosine hydroxylase, and tryptophan hydroxylase (Wijemanne and Jankovic, 2015). Thus far, more than 400 autosomal dominant variants in *GCH1* have been reported among DRD patients worldwide, thus accounting for 66% of all monogenic DRD conditions (Wijemanne and Jankovic, 2015; Weissbach et al., 2021). Dystonia is the most frequent motor and initial sign in the majority of patients with autosomal dominant *GCH1* (AD-*GCH1*) patients (Weissbach et al., 2021). Cases involving autosomal recessive DRD are very rare and usually arise from pathogenic variants in the *TH* or *SPR* genes; these are rarely caused by biallelic autosomal recessive *GCH1* (AR-*GCH1*) variants (Weissbach et al., 2021; Wijemanne and Jankovic, 2015). In comparison to AD-*GCH1* patients, those with AR-*GCH1* usually present with a more complex phenotype and are associated with an infantile onset in most cases. Non-motor features, including global developmental delay and motor delay, are common in AR-*GCH1* patients (Weissbach et al., 2021). In addition, patients with AR-*GCH1* variants are usually diagnosed with hyperphenylalaninemia during a newborn screening and characterized by the neonatal onset of poor sucking and swallowing difficulties, severe hypotonia, seizures, and psychomotor retardation (Bodzioch et al., 2011; Opladen et al., 2011).

Here, we describe the case of one patient without hyperphenylalaninemia who presented with severe truncal hypotonia and motor developmental regression during infancy. A novel homozygous variant of *GCH1* was identified in this patient.

## Case Presentation

An 18-month-old girl, the first child of non-consanguineous Chinese parents, was born at full-term after a non-eventful pregnancy. Her birth weight was 3,650 g and the Apgar score was 10 at both 1 and 5 min. The patient was breast-fed, without feeding problems, and her development was normal during the first few months of life. She sat independently when she was 6 months-of-age. However, she was unable to sit well at 7 months-of-age. Objective neurological findings were hypotonia and strephexopodia. The patient was admitted to another hospital, where routine investigations were performed, including plasma ammonia and lactate, electroencephalography (EEG), and brain magnet resonance imaging (MRI); all findings were negative. She was diagnosed with cerebral palsy; this was followed by rehabilitation training. However, the patient showed no developmental progress.

At the age of 12 months, she developed an obvious floppy trunk without clear diurnal variations and was referred to our department. Physical examination revealed severe truncal hypotonia, symmetrical hyperreflexia, bilateral extensor plantar responses, and valgus deformity of the right foot. In terms of development, the patient showed poor head control; an inability to sit unsupported for more than 20 s, roll over or crawl; and was unable to produce speech (Supplementary Video S1). No similar cases were detected in the family. Her parents were examined and found to be free of neurological symptoms. Laboratory tests were all normal, including blood count, routine biochemistry, serum lactate, ammonia, and ceruloplasmin. Nerve conduction velocities, EEG, ophthalmological examination, brain-stem auditory-evoked and visual-evoked potential studies, and brain MRI were normal. Analysis of plasma amino acids revealed a normal level of phenylalanine while urinary organic acids were also normal.

When referred to us, the patient was suspected to have dopa-responsive dystonia. However, her parents refused to perform genetic analysis along with neurotransmitter and GTPCH-1 enzyme activity analysis. We commenced a trial replacement therapy with low doses of levodopa/carbidopa (1 mg/kg/day). The patient showed an improvement of trunk hypotonia 2 days after starting therapy and was able to sit unsupported for longer than 1 min. Four weeks later, she became able to crawl, control her head, and sit without any support (Supplementary Video S2). Shortly afterwards, she started imitating syllables and began to walk, although slight trunk hypotonia remained. Then, the levodopa/carbidopa dosage was slowly increased, without any significant side effects. Folic acid (5 mg/day) was added to prevent the depletion of cerebral folate by levodopa/carbidopa treatment. The patient showed a gradual, steady, and remarkable improvement in motor function. When the patient was 18 months-of-age (levodopa/carbidopa, 10 mg/kg/day), after she had undergone therapy for 6 months, no hypotonia was observed, and her neurological development was age appropriate (Supplementary Video S3).

**FIGURE 1:**
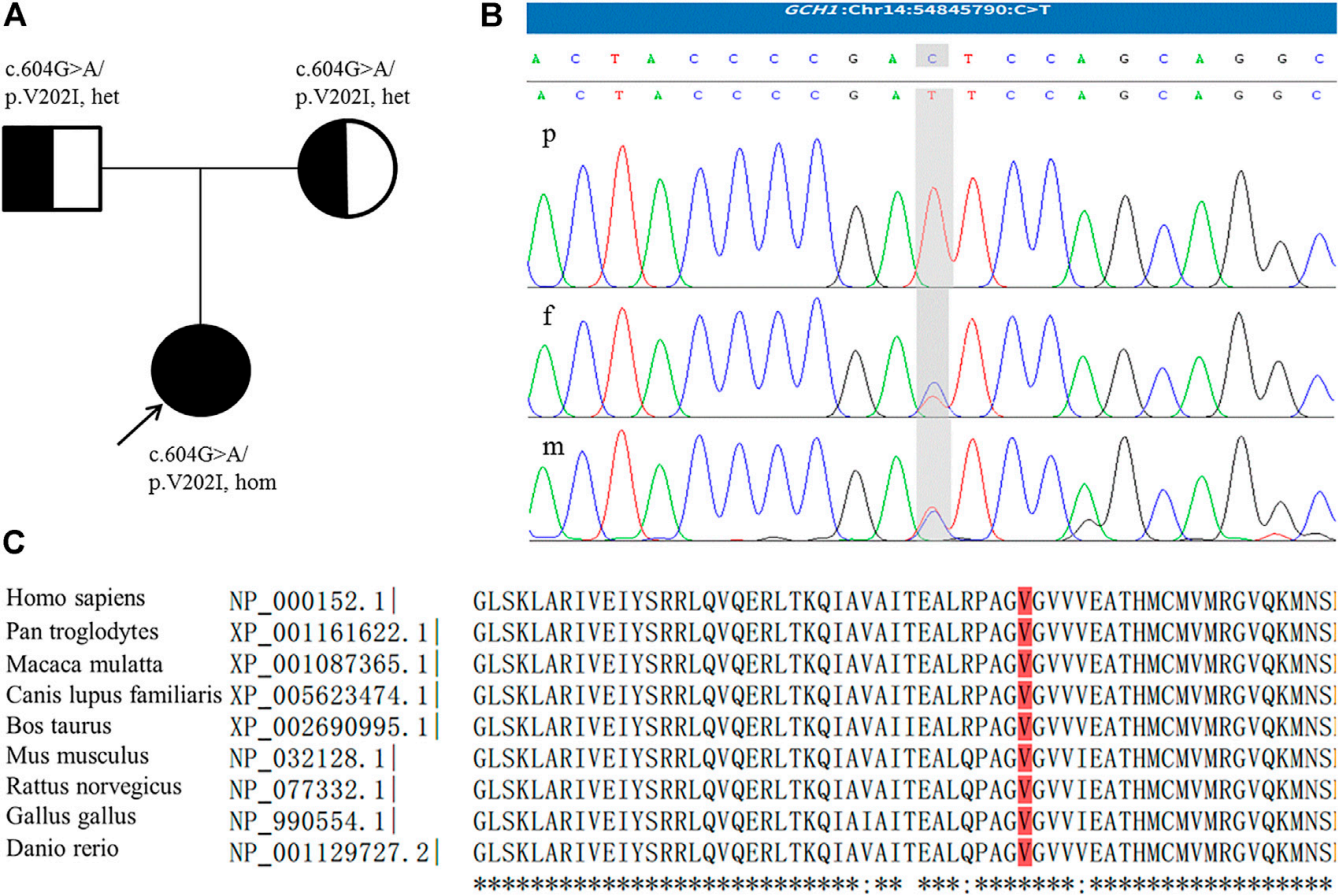
**(A)** Family pedigree; the arrow indicates the proband. **(B)** The homozygous *GCH1* c.604G > A variant detected in the patient (p); the parents were heterozygous for this mutation (f,m). The figure shows reverse strand verification. **(C)** The conserved reside corresponding to the site of the missense variant is shown in red.

During follow-up, genetic analysis was performed after obtaining informed consent from the parents. In accordance with the Declaration of Helsinki of the World Medical Association, genomic DNA from the patient and her parents was extracted from peripheral leukocytes using standard techniques. Exome sequencing of the patient and parents was then performed and targeted variants were verified by Sanger sequencing. The patient was found to be homozygous for a missense variant in *GCH1*, c.604G>A/p.V202I [NM_000161.3] in exon 5. Both parents, who were asymptomatic, were heterozygous for this mutation (Figure 1). This variant is rare, with an allele frequency of 0.0000069773 in the gnomAD database, and is associated with several computational predictors for deleterious conditions. The variant reported was classified as a variant of uncertain significance (VUS) according to the American College of Medical Genetics and Genomics guidelines (PM2_Supporting + PP3) (Richards et al., 2015). However, following a review of the relevant literature, we found a heterozygous p.V202I variant which had been described previously by Hagenah et al. (2005) in a DRD patient with lower limb dystonia. Moreover, several studies have reported homozygous variants associated with DRD presenting with significant hypotonia and limbs dystonia, but without diurnal fluctuation (Nardocci et al., 2003; Opladen et al., 2011; Ray et al., 2022). As a result, we finally ranked the variant carried by our patient as a likely pathogenic variant.

## Literature Review

We searched the PubMed database (https://pubmed.ncbi.nlm.nih.gov/) for homozygous *GCH1* variants by applying search terms in the English literature until March 2022 (for a description of the detailed search strategy, please refer to Supplementary Material S1). We focused on the following information: age at onset, initial symptoms, diagnostic delay, dystonia, diurnal fluctuations of symptoms, other neurological symptoms, and treatment. Our literature search revealed eight articles (Blau et al., 1995; Ichinose et al., 1995; Hwu et al., 1999; Nardocci et al., 2003; Horvath et al., 2008; Opladen et al., 2011; Brüggemann et al., 2012; Ray et al., 2022). We extracted data from 12 patients, including five female and seven male patients. These patients descended from nine families (seven from consanguineous families), and involved nine homozygous variants. Family history was positive in 58% of cases (*n* = 7). Patients showed a median age at onset of 1 month (range: 0–6.5 years) and 83% (*n* = 10) of all patients experienced onset within 1 year. The median diagnostic delay was 8 months (range: 0–1.5 years). Diurnal fluctuation was observed in seven patients (58%). The initial symptoms included abnormal movements in the extremities or developmental delay. Dystonia was the most frequent motor sign and was observed in the limbs (*n* = 12, 100%), trunk (*n* = 9, 75%), and neck (*n* = 7, 58%). Some patients also showed hypotonia (*n* = 6, 50%), or Parkinsonism-likely symptoms (*n* = 8, 67%). Oculogyric crises were reported in five patients (41%). Patients also exhibited deficiency, or partial deficiency, in GTPCH activity (*n* = 8, 100%; four patients were not tested). Several patients also showed reduced levels of homovanillic acid, 5-hydroxyindoleacetic acid, neopterin, and tetrahydrobiopterin (BH4) in cerebrospinal fluid (CSF), as well as hyperphenylalaninemia in the blood (*n* = 3). All patients showed a positive response to levodopa/carbidopa (dose range: 1–20 mg/kg/d). Detailed information is provided in Table 1.

**TABLE 1:** Clinical and genetic features of the current patient and published patients with homozygous *GCH1* variants.

Study	Ichinose et al. (1995)	Blau et al. (1995)	Hwu et al. (1999)		Nardocci et al. (2003)		Horvath et al. (2008)	Opladen et al. (2011)		Brüggemann et al. (2012)		Ray et al. (2022)	Current case
Patient	P1	P2	P3	P4	P5 (twin sister of P4)	P6	P7 (younger brother of P6)	P8	P9	P10 (younger brother of P9)	P11	P12	P13
Mutation	c.551G > A/p.R184H	c.633G > A/p.M211I	c.C747G/p.R249S	c.C595G/p.P199A	c.C595G/p.P199A	c.617T > C/p.V206A	c.617T > C/p.V206A	c.218C > A/p.A73D	c.309G > C/p.Q103H	c.309G > C/p.Q103H	c.703C > G/p.R235G	c.457C > T/p.H153Y	c.604G > A/p.V202I
Sex	F	M	F	F	F	M	M	M	M	M	F	M	F
Age of onset	First week of life	Since birth	2 years and 8 months	<1 month	<1 month	Since birth	Since birth	<3 months	3 months	Prenatal replacement	4 months	6.5 years	7 months
Consanguineous	N	N	N	N	N	Y	Y	Y	Y	Y	Y	Y	N
Family history	N	N	N	Y	Y	Y	Y	Y	Y	Y	N	N	N
Diagnostic delay	6 months	9 months	5 months	12 months	12 months	9 months	<1 month	12 months	7 months	Prenatal diagnosis	4 months	1.5 years	5 months
Clinical features													
Initial symptom or sign	Feeding problems, poor sucking, poor muscle tone	Hypotonia of neck and limbs	Rigidity and tremors of the extremities	Rigidity and tremors of the extremities	Rigidity and tremors of the extremities	Poor suck, tremulous movements	Tremulous movements	Psychomotor development retarded	Jerky leg movements, inability to control the head	Postural tremor of extremities	Delayed milestones	Abnormal walking posturing, tremulousness of upper limbs	Motor developmental regression
Diurnal fluctuation	Y	N	Y	Y	Y	N	N	N	Y	Y	N	Y	N
Limb dystonia	Y	Y	Y	Y	Y	Y	Y	Y	Y	Y	Y	Y	N
Truncal dystonia	NM	Y	Y	Y	Y	Y	Y	Y	Y	Y	NM	NM	Y
Cervical dystonia	NM	Y	Y	Y	Y	N	N	Y	Y	Y	NM	NM	Y
Hypotonia	Y	Y	N	Y	Y	N	N	Y	N	N	Y	N	Y
PLS	Choreoathetosis	PLS movement, tremors	Tremor	Tremor	Tremor	N	N	N	Tremor	Tremor	Choreoathetoid movements	N	N
Oculogyric crises	NM	N	N	N	N	Y	Y	N	Y	Y (suspicious)	Y	N	N
Motor delay	Y	Y	N	Y	Y	Y	Y	Y	Y	N	Y	N	Y
GDD	Y	Y	N	N	N	Y	Y	Y	Y	N	N	N	N
Cognitive impairment	N	Y	N	N	N	N	N	N	Y	N	N	N	N
Microcephaly	N	N	N	N	N	Y	N	N	N	N	Y	N	N
Seizure	Y	N	N	N	N	N	N	N	N	N	N	N	N
Other neurological sign	N	Upper limbs tendon reflexes	Tremor, rigidity	Rigidity, tremor, limbs hyperkinesias, symmetric hyperreflexia	Rigidity, tremor, limbs hyperkinesias, symmetric hyperreflexia	N	N	Rigid; passive flexion was barely possible	Rigidity, tremor, myoclonic jerks; spasticity, brisk tendon reflexes	Tremor, increased muscle tone	N	N	Symmetrical hyperreflexia, bilateral extensor plantar responses
Treatment													
Levodopa/carbidopa	Y	(5.8–15) mg/kg/d	20 mg/kg/d	5 mg/kg/d	5 mg/kg/d	(1–10) mg/kg/d	(1–6) mg/kg/d	(2–8) mg/kg/d	(1–10) mg/kg/d	(1–10) mg/kg/d	1 mg/kg/d	6 mg/kg/d	(4–10) mg/kg/d
BH4	Y	(3.0–3.8) mg/kg/d	N	N	N	2 mg/kg/d	(1–2.5) mg/kg/d	N	N	N	N	N	N
5-HT	Y	(2.3–3.0) mg/kg/d	N	N	N	(1–8) mg/kg/d	(1–4) mg/kg/d	N	N	N	3 mg/kg/d	N	N
Other	N	Low-Phe diet	N	N	N	Folic acid (5 mg/d)	Folic acid (5 mg/d)	N	N	N	Folic acid (15 mg/d), low-Phe diet	Folic acid (10 mg/d)	Folic acid (5 mg/d)
Biochemical features													
HPA, (Phe, normal range) umol/L	Y (>2400, <25)	Y (1488, <20)	N	N	N	N	N	N	N	N	Y (300, 20–150)	N	N
Decrease of CSF HVA, 5HIAA, Neo, BH4	NM	Y (all)	NM	NM	NM	Y (HVA, Neo, BH4)	Y (Neo, BH4)	Y (all)	Y (all)	NM	NM	NM	NM
Plasma Bio and Neo decrease	Y (all)	NM	NM	NM	NM	Y (Bio)	NM	NM	NM	NM	NM	NM	NM
Deficiency of GTPCH-1 activity	Y (liver biopsy not detectable)	Y (liver biopsy not detectable)	Y (mononuclear blood cells, partial deficiency, GTPCH-1 activity 4.2 pmol/mg/h, range: [38.4–102.6] pmol/mg/h)	Y (skin fibroblasts, partial deficiency, GTPCH-1 activity 0.35 uU/mg and 0.36 uU/mg, respectively, range:[1.4–6.5] uU/mg)	Y (skin fibroblasts almost undetectable)	NM	Y (skin fibroblasts, partial deficiency, GTPCH-1 activity reduced down to 35%)	Y (skin fibroblasts, partial deficiency, GTPCH-1 activity reduced down to 17% and 31%, respectively)	Y (skin fibroblasts, partial GTPCH-1 deficiency, activity reduced down to 35%)	NM	NM	NM	
Therapy outcome													
Levodopa-induced dyskinesia	N	N	N	N	N	N	N	N	N	N	N	N	N
Residual symptom	Partial improvement (died at the age of 10-year)	33-month of age: slight ataxic gait, slight mental retardation remained	Completely remission	Both with slight generalized hyperreflexia	Both with slight generalized hyperreflexia	3 years: normal motor and mental development; head circumference improvement	18-month of age: neurological development is age-appropriate)	6 years: average age-related results in all subtests	26-month of age: mental developments delayed, cognitive impairment	17-month of age: started walking, speak single words, mental development delayed	Follow-up 40 months: significant improvement in milestones and dystonia	Follow-up 12 months: baseline milestones was normal, significant improvement in dystonia	Completely remission

M, male; F, female; Y, yes; N, no; NM, not mentioned; PLS, Parkinsonism-likely symptom; HPA, hyperphenylalaninemia; CSF, cerebrospinal fluid; HVA, homovanillic acid; 5HIAA, 5-hydroxyindoleacetic acid; Phe, phenylalanine; Neo, neopterin; Bio, biopterin; BH4, tetrahydrobiopterin; GTPCH-1, GTP, cyclohydrolase І

## Discussion

Here, we report a Chinese patient associated with a homozygous *GCH1* variant. The patient presented with infantile onset of severe hypotonia and normal blood phenylalanine level. Diagnosis was performed by clinical evaluation and response to levodopa and was confirmed by genetic analyses. CSF neurotransmitter analysis, phenylalanine loading tests and GTPCH-1 enzyme activity tests were not performed because of the lack of parental consent. Her symptoms were completely and persistently responsive to levodopa/carbidopa treatment, and without any side effects. Our patient differed from the classic phenotype of the disease: she presented with severe truncal hypotonia and motor developmental regression but without apparent limb dystonia. This case expands the spectrum of DRD phenotypes.

Dopa-responsive dystonia (DRD) encompasses a group of clinically and genetically heterogeneous disorders that typically manifest as limb-onset, diurnally fluctuating dystonia and exhibit a sustained response to levodopa treatment (Wijemanne and Jankovic, 2015). Autosomal dominant GTP cyclohydrolase 1 (GTPCH-1) deficiency, also known as Segawa disease, is caused by dominant *GCH1* variants and is the most common and best-characterized condition that manifests as DRD. Although DRD is usually caused by a dominant variant in *GCH1*, several recessive variants in *GCH1* have also been reported. In DRD patients with recessive *GCH1* variants, there is a spectrum of symptom severities ranging from mild to very severe. Patients with recessive *GCH1* variants could have symptoms of intellectual disability, seizures, abnormal muscle tone, and movements (Blau et al., 1995; Ichinose et al., 1995; Furukawa et al., 1998; Nardocci et al., 2003). However, a patient with a homozygous variant of *GCH1* may manifest with mild symptoms of typical DRD (Hwu et al., 1999). These conditions may be associated with residual GTPCH-1 activities. In cases of patients with recessive variants, the analysis of GTPCH-1 activity in fibroblasts or liver biopsy usually reveals a reduction in activity by up to 35% (Blau et al., 1995; Nardocci et al., 2003; Horvath et al., 2008; Opladen et al., 2011). In patients carrying homozygous variants, the levels of activity may even decrease to far less than 50% (even undetectable) due to the inactivation of both alleles (Blau et al., 1995; Ichinose et al., 1995; Horvath et al., 2008), which might cause more severe symptoms such as severe intellectual disability and seizures. When variants partially affect the expression levels of the enzyme (e.g., 50%), DRD patients with recessive variants might exhibit typical DRD symptoms, as shown in patients with a single dominant variant. In addition, the phenylalanine/tyrosine ratio is usually elevated during the phenylalanine loading test, with a striking reduction of plasma biopterin (Opladen et al., 2011).

In early childhood, the required doses of levodopa for patients with AR-DRD are usually higher than in classical DRD but may decrease with increasing age (Horvath et al., 2008; Bodzioch et al., 2011; Opladen et al., 2011). In patients with compound heterozygosity for *GCH1* mutations, a higher frequency of intolerable levodopa-induced dyskinesias may restrain therapeutic efforts to optimally alleviate extrapyramidal signs (Furukawa et al., 2004; Bodzioch et al., 2011). These dyskinesias in DRD patients subside after dose reduction and do not reappear with subsequent slow dose increments (Kim et al., 2016). However, to date, none of the patients reported to have homozygous *GCH1* variants have exhibited levodopa-induced dyskinesias. In our patient, treatment with levodopa/carbidopa had a dramatic and immediate effect without any side effects. The dosage of levodopa used corresponds to those published for other forms of recessive GTPCH deficiency (Kim et al., 2016; Weissbach et al., 2021). Interestingly, the heterozygous p.V202I variant was first described by Hagenah et al. in a DRD patient with lower limb dystonia (Hagenah et al., 2005). The parents of our patient were found to carry the heterozygous p.V202I variant, but both were asymptomatic and without obvious neurological signs. According to previous reports, approximately 30–50% of individuals with autosomal dominant DRD have no family history of clinically apparent dystonia (Bandmann et al., 1996). Therefore, the possibility that the symptoms associated with a variant of p.V202I with incomplete penetrance cannot be excluded (Bandmann et al., 1996).

Within the broad spectrum of phenotypes, AR-DRD shows some overlapping features with several disorders and is subject to frequent misdiagnosis, particularly as cerebral palsy (Talvik et al., 2010; Flotats-Bastardas et al., 2018; Giri et al., 2019) or epilepsy (Brüggemann et al., 2012; Gowda et al., 2019). Our patient was misdiagnosed as cerebral palsy. Moreover, several reports indicated that mild intellectual disability and/or cognitive impairment may remain, despite long-term treatment. Previous studies reported that slight ataxic gait, intellectual disability, and speech delays remained following the initiation of levodopa/carbidopa for 17–24 months (Blau et al., 1995; Brüggemann et al., 2012); the other cases also experienced neurological impairments, including learning difficulties and moving disorders after 36 months (Bandmann et al., 1998). These reports indicate that early diagnosis and the initiation of replacement treatment during early infancy are important for the prevention of mental and/or cognitive impairment, speech, and movement deficits (Brüggemann et al., 2012).

In terms of the current patient, we were unable to measure pterins levels in the urine and CSF or GTPCH-1 activity in the patient’s fibroblasts cells. Such tests are highly specialized and not readily available; furthermore, we had no parental consent for lumbar puncture. After careful clinical evaluation, we initiated a trial replacement therapy with low doses of levodopa/carbidopa. Shortly afterwards, genetic detection was performed. Fortunately, her symptoms were completely and persistently responsive to levodopa/carbidopa treatment without any side effects and there were no delays in the initiation of treatment and diagnosis.

## Conclusion

We describe one DRD patient associated with a homozygous *GCH1* variant. The patient presented with severe hypotonia, motor developmental regression, without apparent limb dystonia and hyperphenylalaninemia. Autosomal recessive DRD caused by *GCH1* variants should be considered in patients with early infantile onset hypotonia and dystonia, even if diurnal fluctuation and hyperphenylalaninemia are absent. Confirmation of the diagnosis can be achieved by genetic analysis of the *GCH1* gene or by enzyme analysis in cultured skin fibroblasts. The early initiation of levodopa/carbidopa leads to a very favorable long-term outcome regarding motor and mental development.

## Data Availability

The datasets for this article are not publicly available due to concerns regarding participant/patient anonymity. Requests to access the datasets should be directed to the corresponding author.

## Supplementary Material

Supplementary Video S1 Patient at the age of 12 months before starting levodopa/carbidopa therapy: note appropriate social contact, inability to control her head and inability to sit without support.

Supplementary Video S2 Four weeks after the start of levodopa/carbidopa treatment (1 mg/kg/d), the patient was able to control her head and sit without support.

Supplementary Video S3 Six months after commencing of levodopa/carbidopa treatment (10 mg/kg/d), the patient was able to walk; her language was restricted to two-to-three-word sentences. Coordination and neurological development were age appropriate.

Supplementary Material S1 Search strategy for the literature review.
