# Supplementary material for: Case Report: Severe Hypotonia Without Hyperphenylalaninemia Caused by a Homozygous GCH1 Variant: A Case Report and Literature Review
Source: Front Genet. 2022 Jul 13;13:929069. doi: 10.3389/fgene.2022.929069 (PMC9532011; doi:10.3389/fgene.2022.929069)
Supplement: Supplementary file 2 [file Table1.DOCX]

**Table 1. The clinical and genetic features of the current patient and published patients with homozygous *GCH1* variants**

| **Study** | **Ichinose, et al** | **Blau, et al** | **Hwu, et al** | **Nardocci, et al** | | **Horvath, et al** | | **Opladen, et al** | **Brüggemann, et al** | | **Ray, et al** | | **Current case** |
| --- | --- | --- | --- | --- | --- | --- | --- | --- | --- | --- | --- | --- | --- |
| **Patient** | P1 | P2 | P3 | P4 | P5 (twin sister of P4) | P6 | P7 (younger brother of P6) | P8 | P9 | P10 (younger brother of P9) | P11 | P12 | P13 |
| **Mutation** | c.551G>A/p.R184H | c.633G>A/p.M211I | c.C747G/p.R249S | c.C595G/p.P199A | c.C595G/p.P199A | c.617T>C/p.V206A | c.617T>C/p.V206A | c.218C>A/p.A73D | c.309G>C/p.Q103H | c.309G>C/p.Q103H | c.703C>G/p.R235G | c.457C>T/p.H153Y | c.604G>A/p.V202I |
| **Sex** | F | M | F | F | F | M | M | M | M | M | F | M | F |
| **Age of onset** | First week of life | Since birth | 2 years and 8 months | <1 month | <1 month | Since birth | Since birth | <3 months | 3 months | Prenatal replacement | 4 months | 6.5 years | 7 months |
| **Consanguineous** | N | N | N | N | N | Y | Y | Y | Y | Y | Y | Y | N |
| **Family history** | N | N | N | Y | Y | Y | Y | Y | Y | Y | N | N | N |
| **Diagnostic delay** | 6 months | 9 months | 5 months | 12 months | 12 months | 9 months | <1 month | 12 months | 7 months | Prenatal diagnosis | 4 months | 1.5 years | 5 months |
| **Clinical features** | | | | | | | | | | | | | |
| Initial symptom or sign | Feeding problems, poor sucking, poor muscle tone | Hypotonia of neck and limbs | Rigidity and tremors of the extremities | Rigidity and tremors of the extremities | Rigidity and tremors of the extremities | Poor suck, tremulous movements | Tremulous movements | Psychomotor development retarded | Jerky leg movements, inability to control the head | Postural tremor of extremities | Delayed milestones | Abnormal walking posturing, tremulousness of upper limbs | Motor developmental regression |
| Diurnal fluctuation | Y | N | Y | Y | Y | N | N | N | Y | Y | N | Y | N |
| Limbs dystonia | Y | Y | Y | Y | Y | Y | Y | Y | Y | Y | Y | Y | N |
| Truncal dystonia | NM | Y | Y | Y | Y | Y | Y | Y | Y | Y | NM | NM | Y |
| Cervical dystonia | NM | Y | Y | Y | Y | N | N | Y | Y | Y | NM | NM | Y |
| Hypotonia | Y | Y | N | Y | Y | N | N | Y | N | N | Y | N | Y |
| PLS | Choreoathetosis | PLS movement, tremors | Tremor | Tremor | Tremor | N | N | N | Tremor | Tremor | Choreoathetoid movements | N | N |
| Oculogyric crises | NM | N | N | N | N | Y | Y | N | Y | Y (suspicious) | Y | N | N |
| Motor delay | Y | Y | N | Y | Y | Y | Y | Y | Y | N | Y | N | Y |
| GDD | Y | Y | N | N | N | Y | Y | Y | Y | N | N | N | N |
| Cognitive impairment | N | Y | N | N | N | N | N | N | Y | N | N | N | N |
| Microcephaly | N | N | N | N | N | Y | N | N | N | N | Y | N | N |
| Seizure | Y | N | N | N | N | N | N | N | N | N | N | N | N |
| Other neurological sign | N | Upper limbs tendon reflexes | Tremor, rigidity | Rigidity, tremor, limbs hyperkinesias, symmetric hyperreflexia | Rigidity, tremor, limbs hyperkinesias, symmetric hyperreflexia | N | N | Rigid; passive flexion was barely possible | Rigidity, tremor, myoclonic jerks; spasticity, brisk tendon reflexes | Tremor, increased muscle tone | N | N | Symmetrical hyperreflexia, bilateral extensor plantar responses |
| **Treatment** | | | | | | | | | | | | | |
| Levodopa/carbidopa | Y | (5.8-15) mg/kg/d | 20 mg/kg/d | 5 mg/kg/d | 5 mg/kg/d | (1-10) mg/kg/d | (1-6) mg/kg/d | (2-8) mg/kg/d | (1-10) mg/kg/d | (1-10) mg/kg/d | 1 mg/kg/d | 6 mg/kg/d | (4-10) mg/kg/d |
| BH4 | Y | (3.0-3.8) mg/kg/d | N | N | N | 2 mg/kg/d | (1-2.5) mg/kg/d | N | N | N | N | N | N |
| 5-HT | Y | (2.3-3.0) mg/kg/d | N | N | N | (1-8) mg/kg/d | (1-4) mg/kg/d | N | N | N | 3 mg/kg/d | N | N |
| Other | N | Low-Phe diet | N | N | N | Folic acid (5 mg/d) | Folic acid (5 mg/d) | N | N | N | Folic acid (15 mg/d), low-Phe diet | Folic acid (10 mg/d) | Folic acid (5 mg/d) |
| **Biochemical features** | | | | | | | | | | | | | |
| HPA, (Phe, normal range) umol/L | Y (>2400, <25) | Y (1488, <20) | N | N | N | N | N | N | N | N | Y (300, 20-150) | N | N |
| Decrease of CSF HVA, 5HIAA, Neo, BH4 | NM | Y (all) | NM | NM | NM | Y (HVA, Neo, BH4) | Y (Neo, BH4) | Y (all) | Y (all) | NM | NM | NM | NM |
| Plasma Bio and Neo decrease | Y (all) | NM | NM | NM | NM | Y (Bio) | NM | NM | NM | NM | NM | NM | NM |
| Deficiency of GTPCH-1 activity | Y (liver biopsy not detectable) | Y (liver biopsy not detectable) | Y (mononuclear blood cells, partial deficiency, GTPCH-1 activity 4.2 pmol/mg/h, range: [38.4-102.6] pmol/mg/h) | Y (skin fibroblasts, partial deficiency, GTPCH-1 activity 0.35 uU/mg and 0.36 uU/mg, respectively, range:[1.4-6.5] uU/mg) | | Y (skin fibroblasts almost undetectable) | NM | Y (skin fibroblasts, partial deficiency, GTPCH-1 activity reduced down to 35%) | Y (skin fibroblasts, partial deficiency, GTPCH-1 activity reduced down to 17% and 31%, respectively) | Y (skin fibroblasts, partial GTPCH-1 deficiency, activity reduced down to 35%) | NM | NM | NM |
| **Therapy outcome** | | | | | | | | | | | | | |
| Levodopa-induced dyskinesia | N | N | N | N | N | N | N | N | N | N | N | N | N |
| Residual symptom | Partial improvement (died at the age of 10-year) | 33-month of age: slight ataxic gait, slight mental retardation remained | Completely remission | Both with slight generalized hyperreflexia | Both with slight generalized hyperreflexia | 3 years: normal motor and mental development; head circumference improvement | 18-month of age: neurological development is age-appreopriate) | 6 years: average age-related results in all subtests | 26-month of age: mental developments delayed, cognitive impairment | 17-month of age: started walking, speak single words, mental development delayed | Follow-up 40 months: significant improvement in milestones and dystonia | Follow-up 12 months: baseline milestones was normal, significant improvement in dystonia | Completely remission |

*Abbreviation: M, male; F, female; Y, yes; N, no; NM, not mentioned; PLS, Parkinsonism-likely symptom; HPA, hyperphenylalaninemia; CSF, cerebrospinal fluid; HVA, homovanillic acid; 5HIAA, 5-hydroxyindoleacetic acid; Phe, phenylalanine; Neo, neopterin; Bio, biopterin; BH4, tetrahydrobiopterin; GTPCH-1, GTP cyclohydrolase І*
