# Supplementary material for: Case Report: Severe Hypotonia Without Hyperphenylalaninemia Caused by a Homozygous GCH1 Variant: A Case Report and Literature Review
Source: Front Genet. 2022 Jul 13;13:929069. doi: 10.3389/fgene.2022.929069 (PMC9532011; doi:10.3389/fgene.2022.929069)
Supplement: Supplementary file 3 [file Table2.DOCX]

**Search strategy for the literature search**

1. **Search terms**

((((((((((((((((((((((((((dopa-responsive) OR (L-DOPA-responsive)) OR (levodopa-responsive)) OR (Segawa)) OR (dystonia)) OR (dystonic)) OR (dyskinesia)) OR (dyskinetic)) OR (GTP cyclohydrolase deficiency)) OR (hyperkinetic)) OR (movement disorder)) OR (parkinson)) OR (paroxysmal movement)) OR (tremor)) OR (myoclon)) OR (chorea)) OR (choreo)) OR (choreatic)) OR (spastic paraplegia)) OR (spastic paraparesis)) OR (HSP)) OR (Strümpell)) OR (ataxia)) OR (ataxic)) OR (cerebellar)) OR (channelopathy)) AND (((((((((((GCH1) OR (GCHI)) OR (GTP-CH-1)) OR (GTPCH1)) OR (GCH)) OR (GTP cyclohydrolase)) OR (DYT14)) OR (DYT5)) OR (DYT5a)) OR (HPABH4B)) OR (14q22)). Until March 2022, in the English literature.

1. **Results of the literature search**

Articles identified by PubMed search: 722

After excluded by title/abstract and full text (autosomal dominant-*GCH1*)

Relevant articles were read and their references were screened for further relevant articles

Excluding duplication of subjects

Articles included for extraction: 8

Total number of included patients: 12

1. **Chinese databases**

We searched the (April, 2022) the Wanfang (<https://www.wanfangdata.com.cn/index.html>) and CNKI (<https://www.cnki.net/>), identified 52 and 38 articles, respectively. However, no patient with homozygous *GCH1* variant was reported.

1. **List of all included publications**

Blau, N., Ichinose, H., Nagatsu, T., Heizmann, C. W., Zacchello, F., and Burlina, A. B. (1995). A missense mutation in a patient with guanosine triphosphate cyclohydrolase I deficiency missed in the newborn screening program. *The Journal of pediatrics*. 126(3), 401-405. doi: 10.1016/S0022-3476(95)70458-2

Brüggemann, N., Spiegler, J., Hellenbroich, Y., Opladen, T., Schneider, S. A., and Stephani, U., et al. (2012). Beneficial prenatal levodopa therapy in autosomal recessive guanosine triphosphate cyclohydrolase 1 deficiency. *Archives of neurology (Chicago)*. 69(8), 1071

Horvath, G. A., Stockler-Ipsiroglu, S. G., Salvarinova-Zivkovic, R., Lillquist, Y. P., Connolly, M., and Hyland, K., et al. (2008). Autosomal recessive GTP cyclohydrolase I deficiency without hyperphenylalaninemia: Evidence of a phenotypic continuum between dominant and recessive forms. *Mol. Genet. Metab.* 94(1), 127-131. doi: 10.1016/j.ymgme.2008.01.003

Hwu, W. L., Wang, P. J., Hsiao, K. J., Wang, T. R., Chiou, Y. W., and Lee, Y. M. (1999). Dopa-responsive dystonia induced by a recessive GTP cyclohydrolase I mutation. *Hum. Genet.* 105(3), 226-230. doi: 10.1007/s004399900115

Ichinose, H., Ohye, T., Matsuda, Y., Hori, T., Blau, N., and Burlina, A., et al. (1995). Characterization of mouse and human GTP cyclohydrolase I genes. Mutations in patients with GTP cyclohydrolase I deficiency. *The Journal of biological chemistry*. 270(17), 10062-10071

Nardocci, N., Zorzi, G., Blau, N., Fernandez Alvarez, E., Sesta, M., and Angelini, L., et al. (2003). Neonatal dopa-responsive extrapyramidal syndrome in twins with recessive GTPCH deficiency. *Neurology*. 60(2), 335-337. doi: 10.1212/01.WNL.0000044049.99690.AD

Opladen, T., Hoffmann, G., Hörster, F., Hinz, A., Neidhardt, K., and Klein, C., et al. (2011). Clinical and biochemical characterization of patients with early infantile onset of autosomal recessive GTP cyclohydrolase I deficiency without hyperphenylalaninemia. *Movement Disord.* 26(1), 157-161. doi: 10.1002/mds.23329

Ray, S., Padmanabha, H., Gowda, V. K., Mahale, R., Christopher, R., and Sreedharan, S., et al. (2022). Disorders of Tetrahydrobiopterin Metabolism: Experience from South India. *Metab. Brain Dis.* 37(3), 743-760. doi: 10.1007/s11011-021-00889-z
